# Co-infection of H9N2 Influenza A Virus and *Escherichia coli* in a BALB/c Mouse Model Aggravates Lung Injury by Synergistic Effects

**DOI:** 10.3389/fmicb.2021.670688

**Published:** 2021-04-21

**Authors:** Song Wang, Ning Jiang, Wenhao Shi, Hang Yin, Xiaojuan Chi, Yanhui Xie, Jingyun Hu, Yanwei Zhang, Huangping Li, Ji-Long Chen

**Affiliations:** Key Laboratory of Fujian-Taiwan Animal Pathogen Biology, College of Animal Sciences, Fujian Agriculture and Forestry University, Fuzhou, China

**Keywords:** influenza A virus, *Escherichia coli*, co-infection, cytokine, nitric oxide synthase 2

## Abstract

Pathogens that cause respiratory diseases in poultry are highly diversified, and co-infections with multiple pathogens are prevalent. The H9N2 strain of avian influenza virus (AIV) and *Escherichia coli* (*E. coli*) are common poultry pathogens that limit the development of the poultry industry. This study aimed to clarify the interaction between these two pathogens and their pathogenic mechanism using a mouse model. Co-infection with H9N2 AIV and *E. coli* significantly increased the mortality rate of mice compared to single viral or bacterial infections. It also led to the development of more severe lung lesions compared to single viral or bacterial infections. Co-infection further causes a storm of cytokines, which aggravates the host’s disease by dysregulating the JAK/STAT/SOCS and ERK1/2 pathways. Moreover, co-infection mutually benefited the virus and the bacteria by increasing their pathogen loads. Importantly, nitric oxide synthase 2 (NOS2) expression was also significantly enhanced by the co-infection. It played a key role in the rapid proliferation of *E. coli* in the presence of the co-infecting H9N2 virus. Therefore, our study underscores the role of NOS2 as a determinant for bacteria growth and illustrates its importance as an additional mechanism that enhances influenza virus-bacteria synergy. It further provides a scientific basis for investigating the synergistic infection mechanism between viruses and bacteria.

## Introduction

Avian influenza viruses (AIVs) belong to genus influenza A virus within the family *Orthomyxoviridae*. They are divided into highly pathogenic avian influenza viruses (HPAIV) and low pathogenic avian influenza viruses (LPAIV) based on their pathogenicity in chickens. HPAIV is restricted to subset of H5 or H7 subtypes that carry a multi-basic cleavage site in their hemagglutinin (HA) protein, whereas LPAIV covers all the remaining subtypes (Bodewes and Kuiken, 2018; Gischke et al., 2020). Currently, the H9N2 subtype is the most widely circulating and destructive LPAIV (Sun and Liu, 2015; Gu et al., 2017). Although its case fatality rate is much lower than HPAIV, it greatly affects the performance of chickens, such as reduced egg production and retarded growth rate, thus causing a significant economic loss to the poultry industry globally. Its persistence in poultry populations also poses high risks for human health (Gu et al., 2017; Pusch and Suarez, 2018). Like other influenza virus strains, H9N2 AIV is prone to mutations (antigenic drift) or reassortments (antigenic shift) in the natural environment (Li et al., 2020). This plays a vital role in the emergence of pandemic strains and increases risk of zoonotic transmission of AIVs from birds to humans. Several zoonotic AIVs such as H5N1, H5N6, H7N9, and H10N8 are all obtained some or all of the “internal” genes from H9N2 AIV (Sun and Liu, 2015; Suttie et al., 2019; Song and Qin, 2020).

H9N2 also has a synergistic effect with multiple pathogens, which further complicates epidemic prevention and control (Umar et al., 2017). Chickens experimentally infected with H9N2 alone show mild clinical signs and no deaths. However, mixed infections often exacerbate H9N2 infection, which results in severe clinical disease with high mortality. Huang et al. (2017) showed that the co-infection of H9N2 AIV and infectious bronchitis virus (IBV) in chickens resulted in more severe lesions than the AIV-infected group and the IBV-infected group. Moreover, co-infection also significantly alleviated the expression level of chicken surfactant protein A (cSP-A), which plays an essential role in antiviral immunity (Huang et al., 2017). There are also many reports of mixed H9N2 AIV and bacterial infections. For example, co-infection of H9N2 AIV with either *Staphylococcus aureus* or *Haemophilus paragallinarum* enhances viral replication in chickens, causing chickens to exhibit more severe clinical signs than those infected with the virus or bacteria alone (Kishida et al., 2004). Similarly, minks co-infected with H9N2 IAV and *Pseudomonas aeruginosa* have exacerbated histopathological lesions in the lung tissue. They also have higher viral loads and delayed bacterial clearance compared to those with single infections (Bo-Shun et al., 2020). Co-infection of H9N2 AIV with *Escherichia coli* (*E. coli*) is the most common combination in the respiratory diseases of poultry, which often complicates the treatment of respiratory diseases, causing significant clinical signs or deaths. Previous studies have demonstrated that infection of chickens with H9N2 AIV increases their susceptibility to secondary *E. coli* infection. Moreover, adhesion of *E. coli* to host cells significantly increases after H9N2 AIV infection (Ma et al., 2018). *E. coli* also increases the virulence of H9N2 AIV and the mortality of infected birds (Barbour et al., 2009; Jaleel et al., 2017; Mosleh et al., 2017).

Co-infection of H9N2 AIV and *E. coli* can cause severe damage to the lungs of poultry. Despite this, the mechanisms underlying the pathogenesis of H9N2 AIV and *E. coli* co-infection remain poorly understood. Herein, we used a mouse model attempting to explore how these two pathogens work together to exacerbate the disease. Although there are limitations regarding the translation of results from mice to chickens, and a chicken model may be more logical for our experiments, but its obvious limits, such as difficulties in obtaining appropriate antibodies against chicken, and high cost of producing transgenic, gene-knockout or knockin animal model to verify the results drove us to select the mouse model. Additionally, human cases infected by H9N2 influenza viruses in poultry occasionally occur and show flu-like symptoms, implying that this subtype influenza virus has a greater opportunity to adapt to humans and acquire the ability of human-to-human transmission. Therefore, investigating pathogenic mechanisms of H9N2 AIV and *E. coli* infection in a mammalian model can also provide helpful insights into the pathogenic potential of novel H9N2 viruses and bacteria infection in humans in the future.

In this study, *in vivo* experiments demonstrated that H9N2 AIV and *E. coli* have a collusive and promotive function in infected mouse models. Co-infection of H9N2 AIV and *E. coli* led to the production of excessive amounts of cytokines, such as inflammatory factors, chemokines, and interferons in the host’s lungs. This caused severe pulmonary disease after viral-bacterial co-infection. Moreover, co-infection promoted bacterial growth by increasing nitric oxide synthase 2 (NOS2) expression. This increased the content of nitrate needed for bacterial metabolism.

## Materials and Methods

### Ethics Statement

The animal protocol used in this study was approved by “the Regulation of College of Animal Sciences, Fujian Agriculture and Forestry University of Research Ethics Committee” (Permit Number PZCASFAFU2017004). All animal experiments were carried out according to the Regulations for the Administration of Affairs Concerning Experimental Animals approved by the State Council of People’s Republic of China.

### Virus, Bacterial Strain, Cells, and Reagents

H9N2 avian influenza virus (A/Chicken/Fujian/MQ01/2015) (S2-like) was isolated in our laboratory and identified by whole-genome sequencing as described previously (Ke et al., 2014). The GenBank accession numbers for the H9N2 eight segments are MT774533-MT774540. The virus was propagated in 9-day-old specific pathogen-free (SPF) chicken embryo as previously described (Wang et al., 2014). *E. coli* O78 was one the most common serogroup identified among the chicken *E. coli* isolates (Circella et al., 2010; Wang et al., 2013), and the *E. coli* O78 standard strain, which was isolated from chicken in Guangdong province of China in 1990, was sourced from the China Veterinary Culture Collection Center (CVCC, Beijing, China) and grown in Luria-Bertani (LB) broth or agar at 37°C. Murine lung epithelial cell line MLE12 and canine kidney epithelial cell line MDCK purchased from the American Type Culture Collection (ATCC, Manassas, VA, United States) were cultured in Dulbecco’s modified Eagle’s medium (DMEM). The medium contained 10% fetal bovine serum (FBS) supplemented with penicillin (100 U/ml) and streptomycin (100 μg/ml).

The following antibodies were used in this study: anti-IκBα (Santa Cruz Biotechnology, Santa Cruz, CA); anti-STAT1 and anti-phospho-STAT1 (Tyr701), anti-STAT3 and anti-phospho-STAT3 (Tyr705), anti-ERK1/2 and anti-phospho-ERK1/2 (Thr202/Tyr204), anti-JNK and anti-phospho-JNK (Thr183/Tyr185), anti-p38 and anti-phospho-p38 (Thr180/Tyr182) (Cell Signaling Technology, Boston, MA, United States); anti-β-actin (TransGen Biotech, China). Anti-IAV NP was obtained as described previously (Wang et al., 2014). The NOS2 inhibitor S-Methylisothiourea Sulfate (SMT) and NO assay kit were purchased from Beyotime Institute of Biotechnology (Jiangsu, China), and the procedures were performed according to the manufacturer’s instructions.

### Adaptation of H9N2 Virus and Bacteria in Mice

Seven-week-old female SPF BALB/c mice were sourced from Shanghai SLAC Laboratory Animal Co., Ltd (Shanghai, China). H9N2 AIV and *E. coli* were then separately adapted in the mouse lungs before co-infection, and these were carried out under enhanced BSL-2 (BSL-2+) conditions.

For virus adaptation, mice were lightly anesthetized with pentobarbital natricum (40–50 mg/kg) and intranasally inoculated with 50 μL (10^6^ EID_50_) of allantoic fluid containing wild-type H9N2 AIV. After 2 days, the mice were euthanized by cervical dislocation and their lungs were harvested, homogenized with ice cold phosphate-buffered saline (PBS), and centrifuged to collect the supernatant at 2,000× gravity for 10 min. 50 μL of the centrifuged supernatant was used as inoculum in the subsequent passage. Three mice were used for infection of each group per passage. After 15 passages, the infected mice died within 5 days postinfection. Then the virus present in the lung homogenate was cloned twice by plaque purification in MDCK cells. The cloned virus was propagated once in 9-day-old SPF chicken embryo, and all eight gene segments of the mouse-adapted virus were sequenced by whole-genome sequencing as described above.

For bacteria adaptation, mice were anesthetized and intranasally inoculated with *E. coli* (10^7^ CFU). The mouse lungs were then harvested after 2 days, homogenized with LB broth, and centrifuged to collect the supernatant. The centrifuged supernatant was diluted and cultured on MacConkey agar plates at 37°C for 24 h. The red colonies on the MacConkey agar plates were inoculated into LB broth, and 10^7^ CFU of *E. coli* was used as inoculum in the subsequent passage. *E. coli* used herein was derived after 3 passages in mouse lungs followed by bacterial purification.

### Mouse Experiments

For body weight and survival rate experiments, forty of the 7-week-old SPF BALB/c mice were randomly divided into four groups on average: H9N2 infection group, *E. coli* infection group, viral-bacterial concurrent infection group, and PBS-inoculated negative control. The mice were lightly anesthetized using pentobarbital natricum (40–50 mg/kg) and intranasally inoculated with H9N2 AIV (1 × 10^4^ PFU) and/or *E. coli* (1.5 × 10^7^ CFU), or mock infected with PBS. From post-infection onward, clinical symptoms, including depression, huddling, ruffled fur, and respiratory distress were monitored and the body weight of mice were recorded daily. The weight change and death of each mouse were recorded for 10 days, or until they displayed severe unrelieved distress or excessive weight loss (25% weight loss from initial body weight), at which time they were considered to be dead, and euthanized by cervical dislocation.

For other mouse experiments, twelve of the 7-week-old SPF BALB/c mice were randomly divided into four groups on average, and intranasally inoculated with H9N2 AIV (1 × 10^4^ PFU) and/or *E. coli* (1.5 × 10^7^ CFU), or mock infected with PBS. After 48 h of infection, all the mice were euthanized by cervical dislocation, and their spleens and livers were aseptically removed and ground to calculate *E. coli* loads, while the lungs were homogenized to determine viral and *E. coli* loads, and prepare protein and RNA samples (see the method below). The samples were stored in a refrigerator at −80°C for further analysis. Data were obtained from three independent experiments.

### Bacterial and Viral Loads in Mouse Tissues

To calculate *E. coli* loads in mouse tissues, the lungs, livers, and spleens of the mice were first weighed separately. Subsequent homogenization of 60 mg of the tissues in 1 mL LB broth was then done with a tissue grinder. The homogenates were then serially diluted and cultured on MacConkey agar plates at 37°C for 24 h. The red colonies on the MacConkey agar plates were counted and calculated by colony-forming unit (CFU).

To determine viral loads in mouse tissues, 60 mg lungs of mice were first weighed and homogenized in 1 mL ice cold PBS using a tissue grinder. The homogenates were then frozen at -80°C and thawed thrice at room temperature to release the virus, followed by centrifugation at 2,000× gravity for 10 min to collect the supernatant. The supernatants were then titrated by plaque assay as previously described (Wang et al., 2016).

### Histopathology

Histopathological analysis was performed as described previously (Wang et al., 2018). In brief, twelve of the 7-week-old SPF BALB/c mice were randomly divided into four groups on average, and intranasally inoculated with H9N2 AIV (1 × 10^4^ PFU) and/or *E. coli* (1.5 × 10^7^ CFU), or mock infected with PBS. The mice were euthanized 48 h after infection and the lungs were harvested. They were then fixed in 4% paraformaldehyde and embedded in paraffin. 5 μm thick lung sections were subsequently cut and mounted on glass slides followed by hematoxylin-eosin (HE) staining. Histological lesions were then detected using a Nikon Eclipse Ti-E microscope (Kobe, Japan).

### Quantitative Real-Time PCR for Quantification of Cytokines and Other Immune-Related Molecules

Total RNA of 60 mg mice tissues was extracted using TRIzol reagent (Invitrogen). The Moloney murine leukemia virus (M-MLV) reverse transcriptase (Promega, Madison, WI, United States) was then used for cDNA synthesis from 2 μg of total RNA. Quantitative real-time PCR was then done using SYBR Premix Ex Taq II (TaKaRa, Tokyo, Japan). The PCR reaction system used 20 μL volume according to manufacturer’s instructions: 10 μL of 2 × SYBR Premix Ex Taq II, 0.8 μL forward primer (10 μM) and 0.8 μL reverse primer (10 μM), 80–100 ng cDNA, and nuclease-free water was supplemented with appropriate volumes to achieve a 20 μL reaction solution. The thermal cycling parameters were as follows: 95°C for 10 s; 40 cycles of 95°C for 5 s, 60°C for 20 s. For each target gene, a standard curve was established by performing a series of dilutions of the cDNA. β-actin was chosen as a reference gene for internal standardization. For quantification, the 2^−ΔΔCT^ methods were used to calculate the relative RNA levels against β-actin. The primer sequences used in this study are listed in Table 1.

**TABLE 1:** List of primers used in this study.

**Gene**	**Forward (5′-3′)**	**Reversed (5′-3′)**
*IL-6*	TTGCCTTCTTGGGACTGATG	TCTGGCTTTGTCTTTCTTGT
*TNF-*α	TCCCCAAAGGGATGAGAAGTTC	TCATACCAGGGTTTGAGCTCAG
*IL-1*β	TGCCACCTTTTGACAGTGATG	CGTCACACACCAGCAGGTTA
*IL-10*	TGCCTGCTCTTACTGACTGG	GCCTGGGGCATCACTTCTAC
*CCL4*	CTAAGGGTTGAAGCTCTGCCA	GCCATTCCTGACTCCACACT
*CXCL1*	CTGGCATATGACCCCTGAAC	ACTGTTGCCACTGACCATTT
*CXCL2*	GCCAAGGGTTGACTTCAAGA	CGCGACTCACTTGTTCAGTA
*XCL1*	GAACTTACAAACCCAGCGGC	TCAGGGTTATCGCTGTGCTG
*CX3CL1*	ACTGAGTCCCCCTCCACTAC	CTACCATTTCCCCCGCCATT
*IFN-*α	TCCTGCCTGAAGGACAGGAAGG	AGGGCTCTCCAGACTTCTGCTCTG
*IFN-*β	ATTTCTCCAGCACTGGGTGG	CCAGGCGTAGCTGTTGTACT
*IFN*-γ	GCACAGTCATTGAAAGCCTA	GCTGTTGCTGAAGAAGGTAG
*IL-28*	CTGCTTGAGAAGGACATGAG	CAGTTGAAACAGGTTGGAGG
*SOCS1*	CTTCTATTGGGGACCCCTGA	ACGGAGTACCGGGTTAAGAG
*SOCS3*	GGAGAGCGGATTCTACTGGA	TAAGCTCTCTTGGGGGTACT
*NOS2*	GTAGACCAAGGTCACAAGCC	GAGAAATGAGGGCACCTAGC
*β-actin*	CTACAATGAGCTGCGTGTGGC	CAGGTCCAGACGCAGGATGGC

### RNA Sequencing

Total RNA was extracted from mouse lung tissue using TRIzol ^®^ Reagent according to the manufacturer’s instructions (Invitrogen). Library construction and RNA sequencing were performed by Shanghai Majorbio Bio-Pharm Biotechnology Co., Ltd (Shanghai, China). Briefly, RNA-seq transcriptome strand library was prepared following TruSeq^TM^ stranded total RNA Kit from Illumina (San Diego, CA, United States) using 5 μg of total RNA. Shortly, ribosomal RNA (rRNA) depletion instead of poly(A) purification was performed by Ribo-Zero Magnetic kit and then fragmented by fragmentation buffer. Then double-stranded cDNA was synthesized using a SuperScript double-stranded cDNA synthesis kit (Invitrogen, Carlsbad, CA, United States) with random hexamer primers, followed by the ligation of multiple indexing adapters to the ends of the double-stranded cDNA. After the libraries were quantified by TBS380, paired-end RNA-seq sequencing library was sequenced with the Illumina HiSeq X ten (2 × 150 bp read length). All raw data for RNA sequencing were deposited into NCBI (GEO accession number GSE164963).

### Western Blotting

Mouse lung tissues were collected, homogenized in ice cold RIPA lysis buffer (Cell Signaling Technology, Beverly, MA, United States) using a tissue grinder, and lysed for 30 min on ice. After adding 2× loading buffer, lysates were boiled for 5 min. Then the prepared samples were separated by sodium dodecyl sulfate-polyacrylamide gel electrophoresis (SDS-PAGE), and transferred onto a nitrocellulose membrane in a wet tank transfer system. Blots were blocked in TBS buffer (10 mM Tris–HCl, pH 7.4, 150 mM NaCl) containing 5% skim milk for 1 h at room temperature, and then probed with primary antibodies as indicated followed by incubation with horseradish peroxidase-conjugated secondary antibody. The protein bands were visualized by chemiluminescence using the FluorChem E System (ProteinSimple, San Jose, CA, United States). Scanning of band intensity was carried out with ImageJ software. The phosphorylation levels of STAT1, STAT3, ERK1/2, JNK, and p38 were normalized to their corresponding total protein and β-action levels.

### Measurement of Nitric Oxide (NO)

MLE12 cells were seeded in three T25 flasks (group 1, group 2, and group 3), and cultured in DMEM without antibiotics. When the cells became 80–90% confluent, group 2 and group 3 were infected with H9N2 AIV (MOI = 1), while group 1 was mock infected with chick embryo allantoic fluid. After 12 h postinfection, *E. coli* was added into the cell supernatants of three groups (MOI = 10). Simultaneously, group 3 was treated with SMT (1 mM), while group 1 and group 2 were treated with vehicle as controls. Then the cell supernatants were collected at indicated times, and the level of NO was detected by Griess reagent using NO Assay Kit (Beyotime Institute of Biotechnology, Jiangsu, China) according to the manufacturer’s instructions.

### Data Analysis

Survival curves were analyzed using the non-parametric log-rank test (GraphPad Prism 5). Data from other experiments were analyzed using the non-parametric Kruskal–Wallis test followed by Dunn’s test for multiple comparisons. The data from three independent experiments were analyzed and presented as the mean ± standard errors of the mean. The level of statistical significance was set at *P* < 0.05.

## Results

### Adaptation of H9N2 AIV and *E. coli* in Mice Results in Increased Virulence

Most of H9N2 influenza viruses isolated from wild and domestic avian species are avirulent for mice. In order to acquire virulence in mice, H9N2 AIV isolated in chicken was blindly passaged in the lungs of BALB/c mice for 15 generations. The adaptation in mice changed virulence of the virus from asymptomatic infection to lethal outcome. Whole genome sequencing analysis revealed that there were nine nucleotide substitutions which resulted in six amino acid substitutions mapped to PB1, PA, HA, NP, NA, and M genes in the mouse-adapted H9N2 strain (Table 2). However, no nucleotide and amino acid substitutions were observed in the PB2 and NS genes. These multiple amino acid substitutions may play a key role in enhancing the virulence of H9N2 AIV in mice. Similarly, *E. coli* O78 strain (avian-origin) was also adapted in BALB/c mice by serial lung-to-lung passages, leading to increased bacterial virulence in mice. Together, mouse-adapted H9N2 AIV and *E. coli* have acquired virulence determining functions, and are suitable for investigating their pathogenic mechanisms in mouse models.

**TABLE 2:** Nucleotide and amino acid differences between the wild-type (WT) H9N2 strain and the mouse-adapted (MA) H9N2 strain.

**Segments**	**Nucleotide sites**	**WT**	**MA**	**Amino acid sites**	**WT**	**MA**
PB1	2131	A	G	711	I	V
PA	1051	G	A	351	E	K
HA	611	C	A	204	T	K
NP	1131	T	C	377	N	N
NP	1223	T	C	408	V	A
NP	1296	T	C	432	N	N
NA	93	G	A	31	T	T
NA	202	A	G	68	T	A
M	827	T	C	276	F	S

### Co-infection of H9N2 AIV and *E. coli* Increases Mortality and Aggravates Acute Lung Injury

**FIGURE 1:**
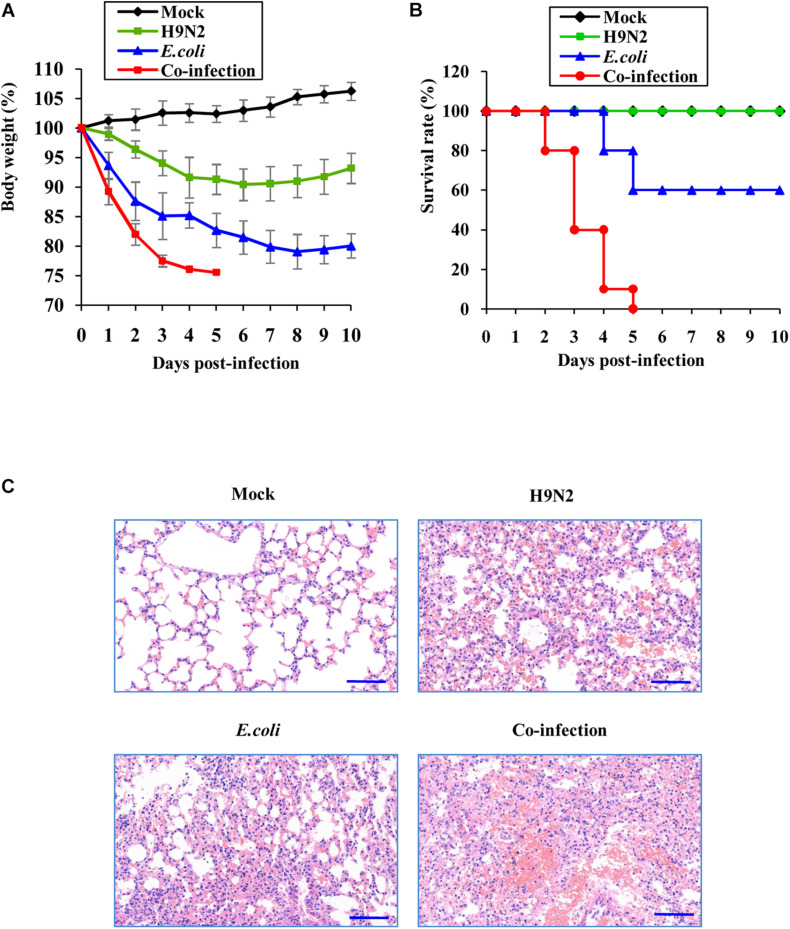
Co-infection of H9N2 AIV and *E. coli* increases mortality and aggravates acute lung injury. Seven-week-old female SPF BALB/c mice were mock infected, or intranasally infected with single H9N2 AIV (1 × 10^4^ PFU) and *E. coli* (1.5 × 10^7^ CFU), or co-infected with H9N2 and *E. coli* for indicated times (*n* = 10 mice/group). **(A)** The body weight changes of mice in four groups. The results are shown as mean percentage weight changes from three independent experiments. **(B)** The survival rate of mice in four groups (*n* = 10). Mice were monitored for up to 10 days. During this period, mice were sacrificed when they displayed severe unrelieved distress or excessive weight loss (25% weight loss from initial body weight). **(C)** Representative images (magnification, ×200) of mouse lungs in four groups after 48 h of infection followed by hematoxylin and eosin (HE) staining.

The mouse model was then used to determine the pathogenic mechanisms of H9N2 AIV and *E. coli*. The weight loss kinetics and survival rate revealed that mice co-infected with H9N2 AIV and *E. coli* had a fast and constant body weight loss (Figure 1A). All the mice succumbed to the co-infection in 5 days. However, the single infection groups had lower fatality rates. There were only 40% deaths in the *E. coli* single infection group and zero deaths in the H9N2 single infection group at the end of the experiments (at day 10 post-infection) under our experimental conditions (Figure 1B).

The pathological changes of mouse lungs were examined after co-infection and single infections to verify the more damaging effects of viral-bacterial co-infection. H9N2 or *E. coli* infected mouse lungs presented as interstitial pneumonia and moderate tissue hemorrhage. However, lung lesions progressed to severe interstitial pneumonia with thickened alveolar walls, and severe alveolar collapse was observed accompanied by large areas of congestion and tissue hemorrhage in the lungs of co-infected mice (Figure 1C). These results indicated that co-infection caused more severe lung damage and increased mortality compared to single infections by the virus or bacteria.

### Cytokine Expressions Are Highly Upregulated in Co-infected Mice

**FIGURE 2:**
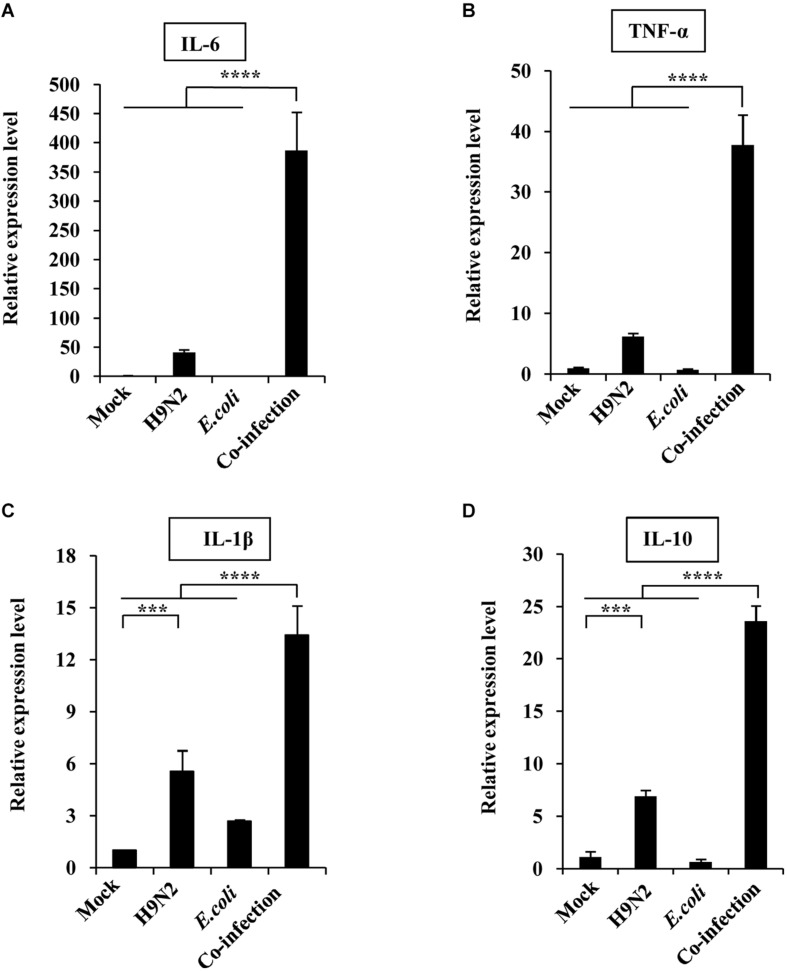
Viral-bacterial co-infection induces excessive expression of inflammatory factors. Four groups of BALB/c mice were mock infected, or intranasally infected with single H9N2 AIV (1 × 10^4^ PFU) and *E. coli* (1.5 × 10^7^ CFU), or co-infected with H9N2 and *E. coli* for 48 h. Quantitative real-time PCR was used to measure the expression of IL-6 **(A)**, TNF-α **(B)**, IL-1β **(C)**, and IL-10 **(D)** in mouse lungs. Data are presented as the mean ± SD of three independent experiments. ***P < 0.001, ****P < 0.0001.

The expression levels of a panel of inflammatory cytokines, chemokines, and interferons in mouse lungs between the co-infection group and the single infection groups were compared. The quantitative real-time PCR results showed that there were no significant differences in the expression levels of inflammatory factors including IL-6, TNF-α, IL-1β, and IL-10 in the lungs of mock-infected mice and those infected with *E. coli* only (Figure 2). However, H9N2-infected mice had a significant increase in inflammatory factors compared to the mock controls. Meanwhile, the co-infected group had exacerbated levels of these inflammatory factors (Figure 2).

**FIGURE 3:**
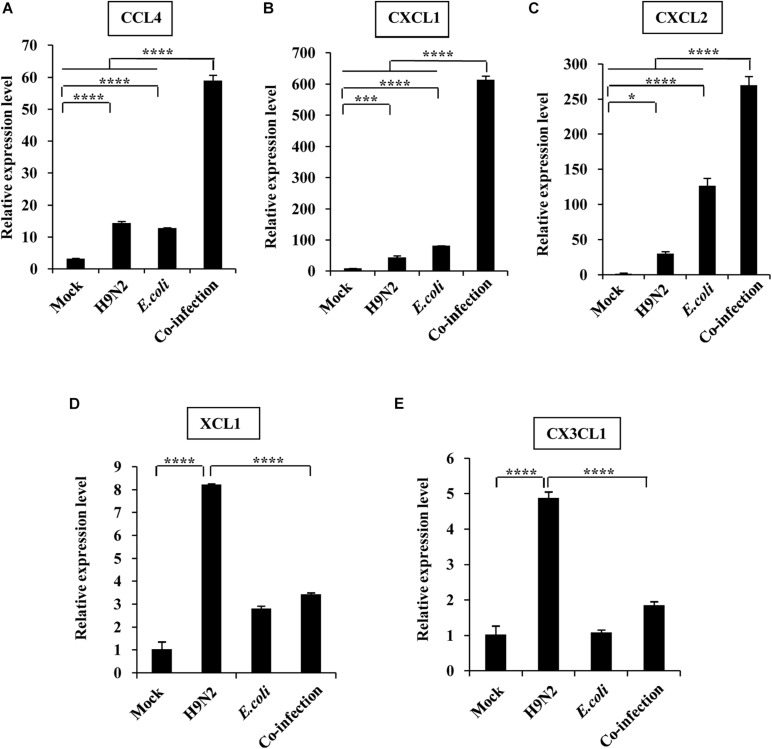
Viral-bacterial co-infection induces aberrant expression of chemokines. Four groups of BALB/c mice were mock infected, or intranasally infected with single H9N2 AIV (1 × 10^4^ PFU) and *E. coli* (1.5 × 10^7^ CFU), or co-infected with H9N2 and *E. coli* for 48 h. Quantitative real-time PCR was used to measure the expression of CCL4 **(A)**, CXCL1 **(B)**, CXCL2 **(C)**, XCL1 **(D)**, and CX3CL1 **(E)** in mouse lungs. Data are presented as the mean ± SD of three independent experiments. *P < 0.05, ***P < 0.001, ****P < 0.0001.

Further, the expression of individual members within four chemokine subfamilies were examined. We found that the expression of CX3C subfamily (CX3CL1) and the C subfamily member (XCL1) was only highly increased in the H9N2-infected mice compared to the mock control, but the expression of CCL4, CXCL1, and CXCL2 was significantly increased in both single infection groups. In addition, CCL4, CXCL1, and CXCL2 expression in the co-infection group was highly significant, while CX3CL1 and XCL1 expression was equivalent to that in mock control (Figure 3).

**FIGURE 4:**
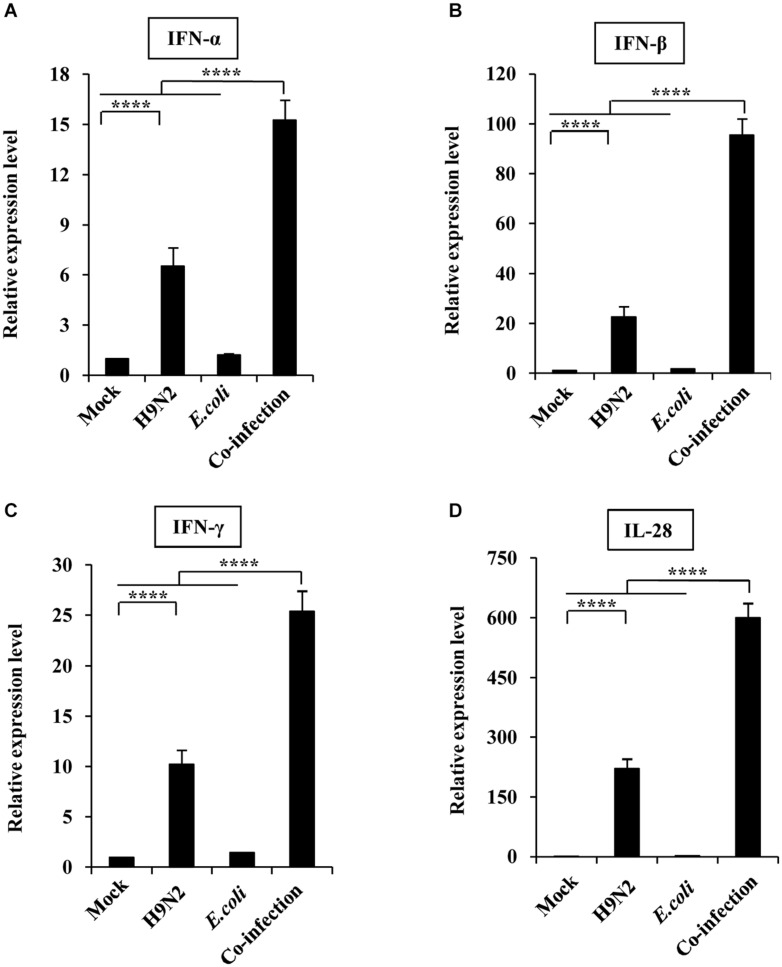
Viral-bacterial co-infection induces aberrant expression of interferons. Four groups of BALB/c mice were mock infected, or intranasally infected with single H9N2 AIV (1 × 10^4^ PFU) and *E. coli* (1.5 × 10^7^ CFU), or co-infected with H9N2 and *E. coli* for 48 h. Quantitative real-time PCR was used to measure the expression of IFNα **(A)**, IFNβ **(B)**, IFNγ **(C)**, and IL-28 **(D)** in mouse lungs. Data are presented as the mean ± SD of three independent experiments. ****P < 0.0001.

The expression levels of type I, II, and III interferons were also measured to determine their role in the severe pathological lung changes in co-infected mice. The expression of IFN-α, IFN-β, IFN-γ, and IL-28 was increased in H9N2-infected mice than that in the mock control. However, the production of the four kinds of interferons was significantly increased in the co-infection group compared to the single infection groups. Among them, the fold change of IFN-β and IL-28 expressions was extremely high (Figure 4). Taken together, these results suggested that H9N2 AIV and *E. coli* co-infection triggered an aberrant immune response, leading to highly upregulated cytokine expressions in mouse lungs.

### JAK/STAT Pathway Works Synergistically With MAPK Pathway to Induce Aberrant Expression of Cytokines After Co-infection With H9N2 and *E. coli*

**FIGURE 5:**
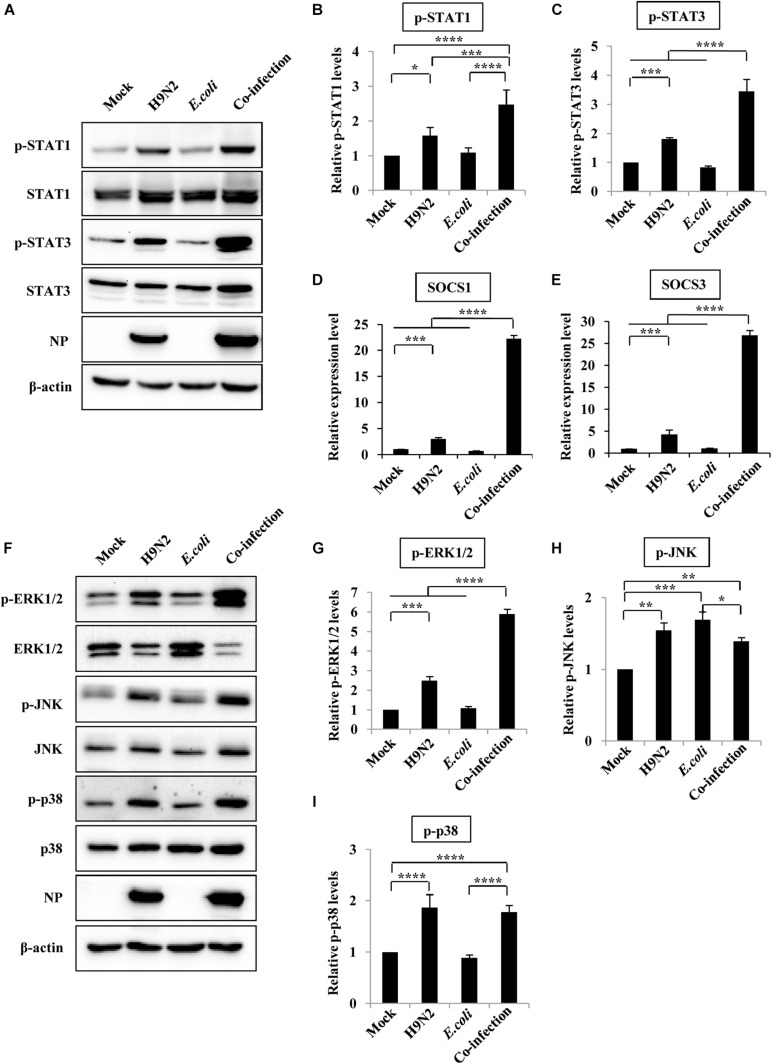
JAK/STAT/SOCS pathway and MAPK pathway are involved in aberrant expression of cytokines after viral-bacterial co-infection. Four groups of BALB/c mice were mock infected, or intranasally infected with single H9N2 AIV (1 × 10^4^ PFU) and *E. coli* (1.5 × 10^7^ CFU), or co-infected with H9N2 and *E. coli* for 48 h. Then the mice were euthanized, and the lungs were ground for the preparation of protein and RNA samples. **(A)** Protein samples were analyzed by western blotting with the indicated antibodies. Shown are representative data of three independent experiments with similar results. **(B,C)** Levels of p-STAT1 **(B)** and p-STAT3 **(C)** in **(A)** were quantitated by densitometry, and normalized to their total protein and β-actin levels. Plotted are the average levels from three independent experiments. The error bars represent the SE. **(D,E)** Quantitative real-time PCR was used for detecting the expression of SOCS1 **(D)** and SOCS3 **(E)** in mice lungs. **(F)** Western blotting was used to determine the phosphorylation level of ERK1/2, JNK, and p38 in mice lungs. The results are representative of three independent experiments. **(G–I)** Levels of p-ERK1/2 **(G)**, p-JNK **(H)** and p-p38 **(I)** in **(F)** were quantitated by densitometry, and normalized to their total protein and β-actin levels. Plotted are the average levels from three independent experiments. The error bars represent the SE. *P < 0.05, **P < 0.01, ***P < 0.001, ****P < 0.0001.

Activation of JAK/STAT, and MAPK signaling pathways, which play a crucial role in cytokine production, was examined to understand why H9N2 and *E. coli* co-infection resulted in an excessive expression of various cytokines. Herein, there was a significant increase in the expression of phosphorylated STAT1 and STAT3 in mouse lungs after H9N2 infection. An even more significant increase in the phosphorylation level of STAT1 and STAT3 was detected in the co-infection group compared to any other group. This was particularly the case for STAT3 (Figures 5A–C).

Activated STAT proteins form dimers and translocate to the nucleus, where they regulate gene expression. SOCS1 and SOCS3 are target genes of STAT1 and STAT3, respectively, and are critical negative regulators of JAK-STAT signaling and thus maintain normal cytokine levels during viral or bacterial infections. Herein, there was a 3 and 4-fold increase in SOCS1 and SOCS3 mRNA expression, respectively, in mice infected with the H9N2 virus alone compared to that of mock-infected controls. However, their expression levels increased 22 and 26-fold, respectively, in co-infected mice (Figures 5D,E).

In addition, MAPKs (ERK1/2, JNK, and p38) also play a significant role in inflammatory responses to pathogen invasion (de Souza et al., 2014; Wu et al., 2018). As such, the phosphorylation level of ERK1/2, JNK, and p38 was measured to determine whether the MAPK signaling pathway was involved in the excessive cytokine expression during co-infection. There was a significant increase in the phosphorylation levels of ERK1/2, JNK, and p38 in the H9N2 AIV infection group compared to the mock-infected control group. However, only the phosphorylation levels of ERK1/2 were significantly increased in the co-infected mice compared to those of any other group (Figures 5F–I). These results suggested that co-infection induced an aberrant expression of cytokines by modulating the STAT1/SOCS1, STAT3/SOCS3, and ERK1/2 signaling pathways.

### H9N2 and *E. coli* Loads Are Increased in Co-infections

**FIGURE 6:**
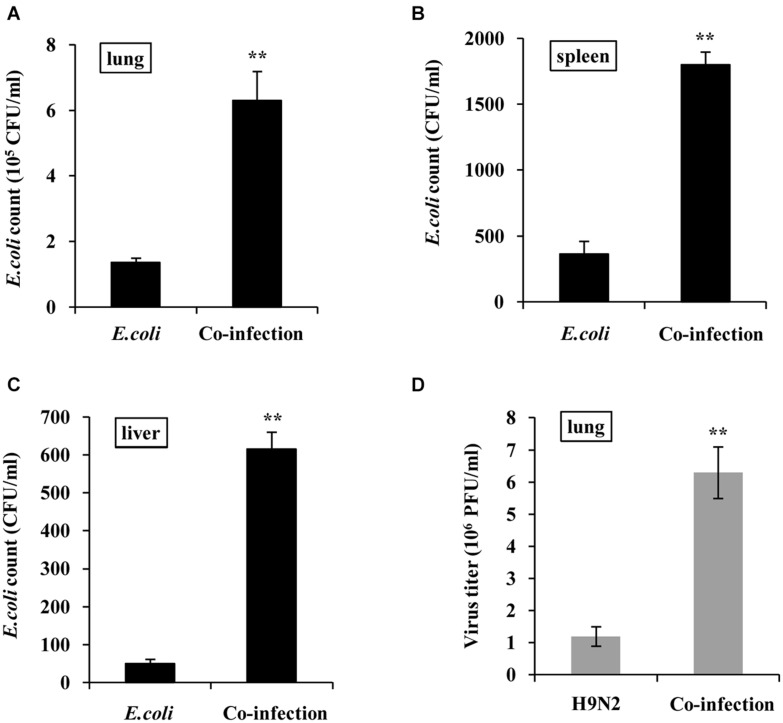
H9N2 and *E. coli* loads are increased in co-infections. BALB/c mice were intranasally infected with single H9N2 AIV (1 × 10^4^ PFU) and *E. coli* (1.5 × 10^7^ CFU), or co-infected with H9N2 and *E. coli* for 48 h. **(A–C)** Bacterial loads in mouse lungs **(A)**, spleens **(B)**, and livers **(C)** were measured. **(D)** Virus titers in mouse lungs were determined by plaque assay. Data are presented as the mean ± SD of three independent experiments. **P < 0.01.

To define the effect of co-infection on viral and bacterial load, mice were infected with H9N2 AIV or *E. coli* alone, or co-infected with these two pathogens for 48h. Results showed that there was a significant increase in bacterial load in the lung, spleen, and liver tissues of co-infected mice compared to that in *E. coli* single infection group (Figures 6A–C). In addition, plaque assay results revealed that the viral load in the lungs of co-infected mice was significantly higher than that in the H9N2 AIV single infection group (Figure 6D). These results indicated that the coexistence of H9N2 AIV and *E. coli* in the mice’s lungs synergistically increased their loads, aggravating the tissue damage of co-infected mice.

### Increased NOS2 Expression Promotes *E. coli* Proliferation

**FIGURE 7:**
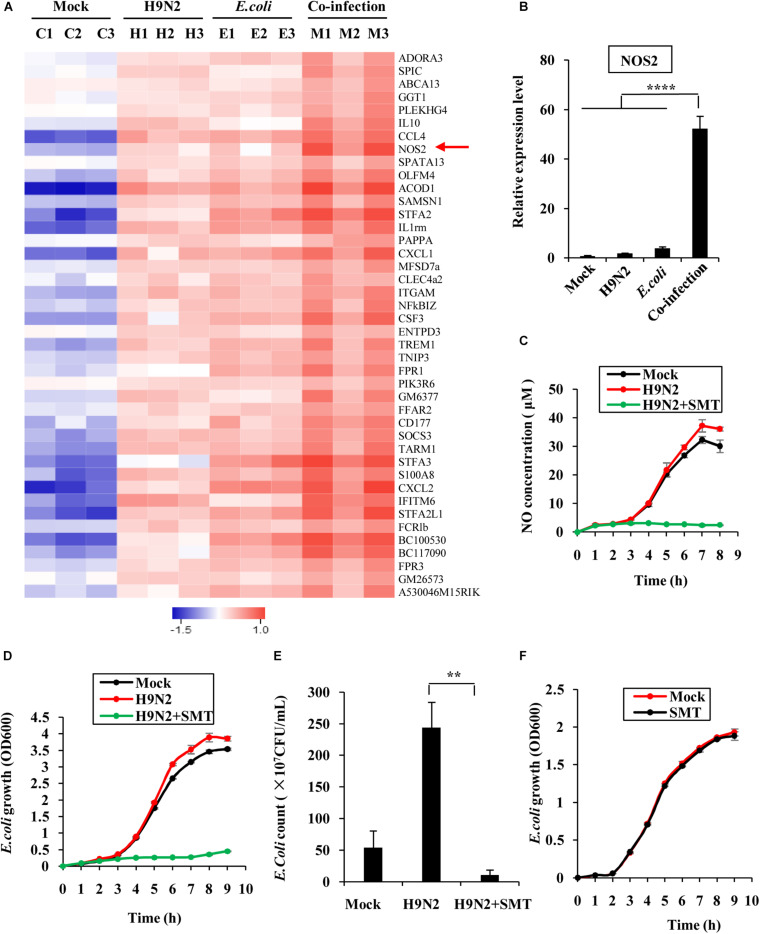
Increased *NOS2* expression promotes *E. coli* proliferation. **(A)** RNA sequencing was used to determine the differentially expressed genes in mouse lungs infected for 48 h between individual infection and co-infection groups. All the genes whose expressions were upregulated and changed by at least 2-fold were displayed. The red arrow highlight the upregulated *NOS2* genes. **(B)** The mRNA expression level of *NOS2* in mouse lungs was examined by quantitative real-time PCR. **(C)** MLE12 cells were infected with or without H9N2 (MOI = 1) for 12 h, followed by *E. coli* (MOI = 10) infection with or without S-Methylisothiourea Sulfate (SMT) treatment. NO production in culture supernatants at the indicated time point was measured by NO assay kit. **(D)** MLE12 cells were infected with or without H9N2 as described in **(C)**. OD_600_ of *E. coli* in cell culture supernatants at the indicated time point was measured by spectrophotometer. **(E)** MLE12 cells were infected with or without H9N2 (MOI = 1) for 12 h, followed by *E. coli* (MOI = 10) infection with or without SMT treatment. After 6 h, the number of *E. coli* in cell culture supernatants was counted. **(F)** *E. coli* cultured in antibiotic-free DMEM was treated with SMT or vehicle control. OD_600_ of *E. coli* in the supernatants at the indicated time point was measured by spectrophotometer. Data are presented as the mean ± SD of three independent experiments. **P < 0.01, ****P < 0.0001.

RNA sequencing was performed to compare the differentially expressed genes in the lungs of co-infected mice and those in the single pathogen infection groups (GEO accession number GSE164963). This was done to explore why H9N2 AIV and *E. coli* had a mutual promotion of the proliferation. There were 42 genes that were significantly upregulated in the co-infection group (Figure 7A). Most of these genes were involved in the inflammatory response, cell chemotaxis, and immune response regulation. Among them, the *NOS2* gene, which encodes inducible nitric oxide synthase, was significantly expressed (Figure 7A). Inducible nitric oxide synthase catalyzes nitric oxide production, which in turn contributes to the proliferation of *E. coli* through nitrate generation (Winter et al., 2013; Baumler and Sperandio, 2016). The mRNA expression levels of *NOS2* in the mice lungs were further examined by quantitative real-time PCR to confirm the RNA sequencing results. Supportively, NOS2 expression in H9N2 virus or *E. coli* single infection group was moderately elevated compared to that of the mock group. However, it was significantly expressed in the co-infection group (Figure 7B).

SMT was further used to block the activity of NOS2 in MLE12 cells to precisely determine the effect of NOS2 expression on *E. coli* proliferation. Co-infected MLE12 cells had a higher concentration of nitric oxide than *E. coli* single-infected cells, suggesting that co-infection increases the expression of NOS2 *in vitro*. However, the addition of SMT into the supernatant of H9N2 virus and *E. coli* co-infected cells significantly decreased nitric oxide production (Figure 7C). Examination of the growth patterns of the bacteria further revealed that it was positively correlated with nitric oxide generation. The bacteria grew faster in the co-infection group than that of the single infection group. The addition of SMT almost completely suppressed the growth of *E. coli* (Figure 7D). Similarly, the number of bacteria was significantly higher in the co-infection group but significantly dropped after the addition of SMT (Figure 7E). To examine whether SMT exerted a direct bacteriostatic effect on *E. coli*, *E. coli* cultured in antibiotic-free DMEM was treated with SMT or vehicle control. There was no significant difference in growth patterns of *E. coli* between the two groups, indicating that SMT could not inhibit *E. coli* growth directly (Figure 7F). These findings confirmed that increased NOS2 expression in the co-infection group promoted proliferation of *E. coli*.

## Discussion

Co-infection of H9N2 AIV and *E. coli* is a common clinical incident associated with significant economic losses to the poultry industry. The H9N2 influenza virus is easy to be ignored or underestimated because chickens experimentally infected with the virus are usually asymptomatic or have mild clinical signs. *E. coli* is a normal intestinal microbial flora, which is also abundant in the breeding environment (Blaak et al., 2015). Co-infection with these two respiratory pathogens can cause severe lesions and increased fatality rate in chickens (Jaleel et al., 2017). This study focused on the interaction between H9N2 AIV and *E. coli* and their impact on the host using a mouse model. BALB/c mice and C57BL/6 mice are two of the most common and convenient mammalian models for studying the pathogenesis of many influenza viruses. However, BALB/c mice are more commonly used as animal model for adaptation of avian influenza virus than C57BL/6 mice (Wang et al., 2012; Li et al., 2014; Kamiki et al., 2018). Therefore, in this study, to make H9N2 AIV and *E. coli* proliferate effectively in mice model, they were separately adapted in BALB/c mice for several lung-to-lung passages, and used to infect their adaptation-performed host in the subsequent experiments. We found that a co-infection of H9N2 avian influenza virus and *E. coli* significantly increased the mortality rate compared to single pathogen infection, and the lung lesions were more severe. These results were similar to those obtained after co-infection of H9N2 avian influenza virus with *Pseudomonas aeruginosa* in mink and co-infection of influenza virus with *Staphylococcus aureus* in patients (McDanel et al., 2016; Bo-Shun et al., 2020).

Lung pathological lesions caused by pathogenic infections are often accompanied by overexpression of various cytokines (Teijaro et al., 2011; Tay et al., 2020). These cytokines play vital roles in the host’s antiviral innate immunity when produced moderately. However, the accumulation of abundant immune cells in the lungs often results in excessive cytokine expression, which leads to severe lung damage and high mortality rates. Herein, co-infection of H9N2 AIV and *E. coli* led to an aberrant expression of cytokines, including inflammatory factors, chemokines, and interferons. Inflammatory factors and interferons are indispensable in the host’s antibacterial and antiviral process. Nonetheless, their aberrant expression is linked to the severity of pneumonia from pathogen infection. Chemokines, which are classified into C, CC, CXC, and CX3C subfamilies according to the NH2-terminal motif of conserved cysteine residues, can mediate leukocyte migration and positioning to the sites for viral or bacterial infection (Baggiolini, 1998; Zargari et al., 2020). In this study, it is revealed that an aberrant chemokine production including CC and CXC subfamily members mediates the recruitment of more leukocytes to the lungs, thus contributing to the induction and progression of lung pathology. This mechanism might be the primary cause of elevated morbidity and mortality.

To understand the underlying mechanism of aberrant cytokine production, some critical transcription factors that were involved in cytokine expression were detected. Different cytokines have the propensity to activate specific STATs. Inflammatory factors such as IL-6, IL-1β, and TNF-α are closely related to STAT3 activation, while interferons induce activation of STAT1 (Villarino et al., 2017). The phosphorylation level of STAT1 and STAT3 was significantly increased after H9N2 and *E. coli* co-infection. This was particularly the case for STAT3. SOCS1 and SOCS3 are negative feedback regulators of STAT1 and STAT3. They initiate a negative feedback loop to keep the balance of host secreted cytokines. Nevertheless, excessive expression of SOCS1 and SOCS3 can significantly increase the production of interferons and inflammatory factors by modulating the function of NF-κB, causing increased disease severity because of overstimulation of the immune system (Wei et al., 2014; Liu et al., 2019). Herein, the expression of SOCS1 and SOCS3 in the lungs of co-infected mice was much higher than that in individual infection groups and the mock group. The expression level of IκBα was then further determined as an indirect indicator of NF-κB activation. However, there was no significant difference in the expression level of IκBα between the co-infection group and single infection groups (Data not shown). This finding suggested that the excessive expression of cytokines did not result from NF-κB activation. This may be explained by the high expression of *Nfkbiz*, which can encode a protein negatively regulating NF-κB activity (Totzke et al., 2006), in co-infection group showed in RNA sequence results.

Expression of SOCS proteins is also associated with activation of the MAPK signaling pathway, which plays a vital role in host inflammation response (Luo et al., 2020). Herein, phosphorylation of ERK1/2, but not JNK and p38, was significantly increased in the co-infection group compared to the single infection groups and mock group. It could have mediated the overproduction of cytokines. However, the regulation mechanism between JAK/STAT/SOCS and ERK1/2 needs further in-depth studies.

Similar types of viruses or bacteria often pose a competitive relationship when they intrude the same host cell (Baumler and Sperandio, 2016). However, virus-bacteria co-infections usually exhibit a synergetic effect (Bo-Shun et al., 2020; Gonzalez-Juarbe et al., 2020; Karwelat et al., 2020; Shi et al., 2020; Wang et al., 2020). In this study, the viral load in the lungs of co-infected mice was significantly higher than that in the H9N2 single infection group. Similarly, the bacterial load in the lung, spleen, and liver of the co-infected mice was significantly higher than that in *E. coli* single infection group. These results confirmed that the influenza virus and *E. coli* worked synergistically during the co-infection process. RNA sequencing further revealed that the expression of *NOS2* was significantly elevated after virus-bacteria co-infection. Its expression was positively correlated with the multiplication of *E. coli*. Previous studies demonstrated that IFN-γ induces NOS2 expression, which in turn triggers the formation of nitrate that’s used by bacteria for growth through nitrate respiration (Winter et al., 2013; Baumler and Sperandio, 2016). The report was consistent with the findings of this study. It further explained why the number of *E. coli* remarkably increased during H9N2 AIV and *E. coli* co-infection. Moreover, previous studies postulated that *Staphylococcus aureus* promotes the replication of influenza viruses by secreting a protease to cleave precursor HA into HA1 and HA2 fragments (Tashiro et al., 1987). Exploration of whether H9N2 and *E. coli* exploited a similar mechanism to achieve synergistic effect revealed that this may be the case, because supplementing the filtered *E. coli* culture medium into H9N2 infected cells promoted the replication of H9N2 AIV (data not shown). Complete genome sequence of *E. coli* O78 showed that it can produce three trypsin-like serine proteases (Mangiamele et al., 2013), which may play the part of influenza virus HA activation like *Staphylococcus aureus* protease does. Construction of serine protease-deletion mutants of *E. coli* will help to demonstrate whether the trypsin-like serine proteases were responsible for the elevated viral load in the co-infected mice. This is our ongoing task.

This study evaluated and discussed the interaction and mutually beneficial cooperation between H9N2 AIV and *E. coli* during co-infection in BALB/c mice. Their synergistic effects play an essential role in host immune responses and disease development. The findings of this study highlight the importance of unraveling the mechanism of virus-bacteria co-infections for developing strategies to treat and control diseases caused by multiple infections.

## Data Availability Statement

The datasets presented in this study can be found in online repositories. The names of the repository/repositories and accession number(s) can be found in the article/supplementary material.

## Ethics Statement

The animal study was reviewed and approved by the College of Animal Sciences, Fujian Agriculture and Forestry University of Research Ethics Committee.

## Author Contributions

SW conceived and designed the experiments. NJ, WS, HY, XC, YX, JH, YZ, and HL performed the experiments and analyzed the data. SW, NJ, and J-LC wrote and revised the manuscript. All authors read and approved the final manuscript.

## Conflict of Interest

The authors declare that the research was conducted in the absence of any commercial or financial relationships that could be construed as a potential conflict of interest.
